# Three-dimensional brain phantom containing bone and grey matter structures with a realistic head contour

**DOI:** 10.1007/s12149-012-0655-7

**Published:** 2012-09-26

**Authors:** Hidehiro Iida, Yuki Hori, Kenji Ishida, Etsuko Imabayashi, Hiroshi Matsuda, Masaaki Takahashi, Hirotaka Maruno, Akihide Yamamoto, Kazuhiro Koshino, Junichiro Enmi, Satoshi Iguchi, Tetsuaki Moriguchi, Hidekazu Kawashima, Tsutomu Zeniya

**Affiliations:** 1 Department of Investigative Radiology, National Cerebral and Cardiovascular Center Research Institute, 5-7-1 Suita City, Osaka, 565-8565 Japan; 2 Saitama Medical University International Medical Center, Hidaka City, Japan; 3 Nakamura Memorial Hospital, Sapporo City, Japan; 4 Toranomon Hospital, Tokyo, Japan

**Keywords:** Brain phantom quality control, SPECT, PET, Attenuation correction

## Abstract

**Introduction:**

A physical 3-dimensional phantom that simulates PET/SPECT images of static regional cerebral blood flow in grey matter with a realistic head contour has been developed. This study examined the feasibility of using this phantom for evaluating PET/SPECT images.

**Methods:**

The phantom was constructed using a transparent, hydrophobic photo-curable polymer with a laser-modelling technique. The phantom was designed to contain the grey matter, the skull, and the trachea spaces filled with a radioactive solution, a bone-equivalent solution of K_2_HPO_4_, and air, respectively. The grey matter and bone compartments were designed to establish the connectivity. A series of experiments was performed to confirm the accuracy and reproducibility of the phantom using X-ray CT, SPECT, and PET.

**Results:**

The total weight was 1997 ± 2 g excluding the inner liquid, and volumes were 563 ± 1 and 306 ± 2 mL, corresponding to the grey matter and bone compartments, respectively. The apparent attenuation coefficient averaged over the whole brain was 0.168 ± 0.006 cm^−1^ for Tc-99 m, which was consistent with the previously reported value for humans (0.168 ± 0.010 cm^−1^). Air bubbles were well removed from both grey-matter and bone compartments, as confirmed by X-ray CT. The phantom was well adapted to experiments using PET and SPECT devices.

**Conclusion:**

The 3-dimensional brain phantom constructed in this study may be of use for evaluating the adequacy of SPECT/PET reconstruction software programs.

## Introduction

SPECT and PET can provide volumetric images of radio-labelled ligands’ distributions in living organs, reflected by biological and/or biochemical functions. Several procedures need to be adequately taken into account, in order to achieve quantitative reconstruction, including corrections for inhomogeneous detector sensitivity, dead time, attenuation and scatter in the object [1–5], motion of the object [6–8], systematic errors attributed to limited spatial resolution of the imaging devices relative to the object size (or partial volume effect, PVE) [9, 10], etc. Adequacy of the entire procedures can be evaluated using physical phantoms that simulate geometrical configurations. The Hoffman 3-dimensional brain phantom [11] has also been utilized for this purpose, as this phantom simulates the static cerebral perfusion of the grey and white matter. The digital design of the cortical grey matter in the phantom is referred as a gold standard of the reconstructed images. Inter-institutional reproducibility is also an issue, as Joshi et al. [12] intended to minimize the inter-institutional variation of images of Hoffman 3-dimensional phantom in a multicentre clinical study using PET devices supplied from different vendors.

The Hoffman 3-dimensional brain phantom is however limited attributed to its cylindrical outer structure rather than a realistic brain contour, and also to the bone or skull structure not being taken into account. These two factors are particularly important if one intends to apply it for evaluating SPECT images, because the attenuation coefficient map is usually estimated from the head contour by assuming a uniform attenuation coefficient value throughout the head object. Effects of errors in this process on errors in the reconstructed images could not be evaluated if the Hoffman 3-dimensional phantom is utilized. It is essential for the phantom to contain a fine 3-dimensional distribution of cerebral radioactivity with the skull and a realistic head contour.

This study was aimed at developing a 3-dimensional brain phantom that simulates a static cerebral blood flow distribution in the grey matter with an inclusion of the skull structure and a realistic head contour. A recently developed photo-curable laser-modelling technique with the lipophilic resin material was employed, so that the phantom contains liquid solutions for radioactivity and the bone-equivalent contrast agent. Attention was made in the construction procedures to avoid the supporting structures, so that the whole volume of the inner spaces of the phantom can be entirely filled with liquid. We also intended to establish the connectivity of liquid space so that air bubbles are to be removed. We then evaluated the feasibility of using this phantom in typical SPECT and PET imaging.

## Materials and methods

### Phantom design and construction

The phantom was made of a transparent photo-curable polymer, or polyepoxide with a density of 1.07 g/mL (TSR-829, CMET Inc., Yokohama City, Japan). This material has been optimized to improve its anti-water absorbing characteristics [13]. According to precise material elements, the database for the photon cross sections (XCOM) from the National Institute of Standards and Technology (http://physics.nist.gov/PhysRefData/Xcom/html/xcom1.html) estimated that the attenuation coefficients for 511, 159, and 140 keV were 0.101, 0.157, and 0.164 cm^−1^, respectively. The phantom was constructed using a stereo-lithographic machine with the laser-modelling technique (model RM-6000, CMET Inc, Yokohama City, Japan), which can produce the 3-dimensional construction in 50 mμ resolution. It was intended not to include any supporting structures in the inner spaces. The manufacturing speed was decreased during the laser-beam construction, so as to ensure the solidity of the phantom, thus avoiding the possible distortion of the inner structure.

**Fig. 1:**
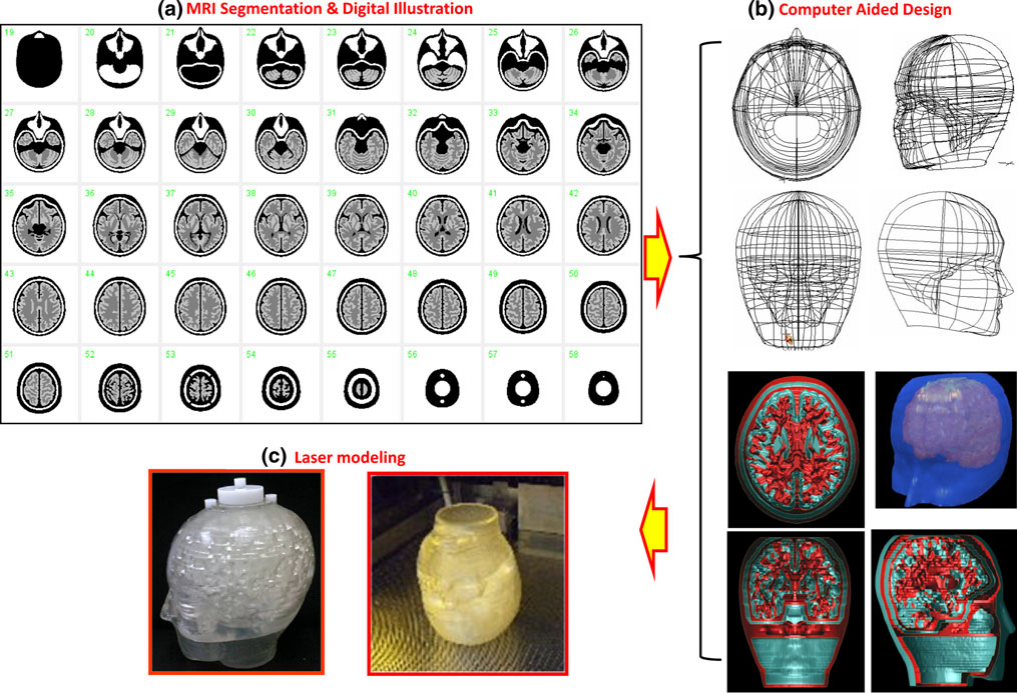
Illustrative procedures to develop the 3-dimensional brain phantom. **a** Tomographic images including the cortical grey matter, deep grey matter, white matter, cerebral spinal fluid space, skull, scalp and trachea regions that were generated at 3.6-mm intervals from anatomical MR images of a young healthy volunteer. Data were manually modified to fit to a head model. **b** Three-dimensional data were then generated using the computer-aided-design software, and modifications were made to guarantee the connectivity of both the grey matter and bone compartments. Careful attention was made to establish a liquid flow stream within the structure so that air bubbles could be easily removed from the liquid space. **c** A laser-modelling technique with a stereo-lithographic machine and a photo-curable material was employed to construct the 3-dimensional phantom. There was special attention to avoid contamination from the resin-based supporting material inside the grey matter and skull compartments, and also to make the inner wall surface of the phantom as smooth as possible. The speed and pitch of the machine as well as the temperature and humidity were optimized

**Fig. 2:**
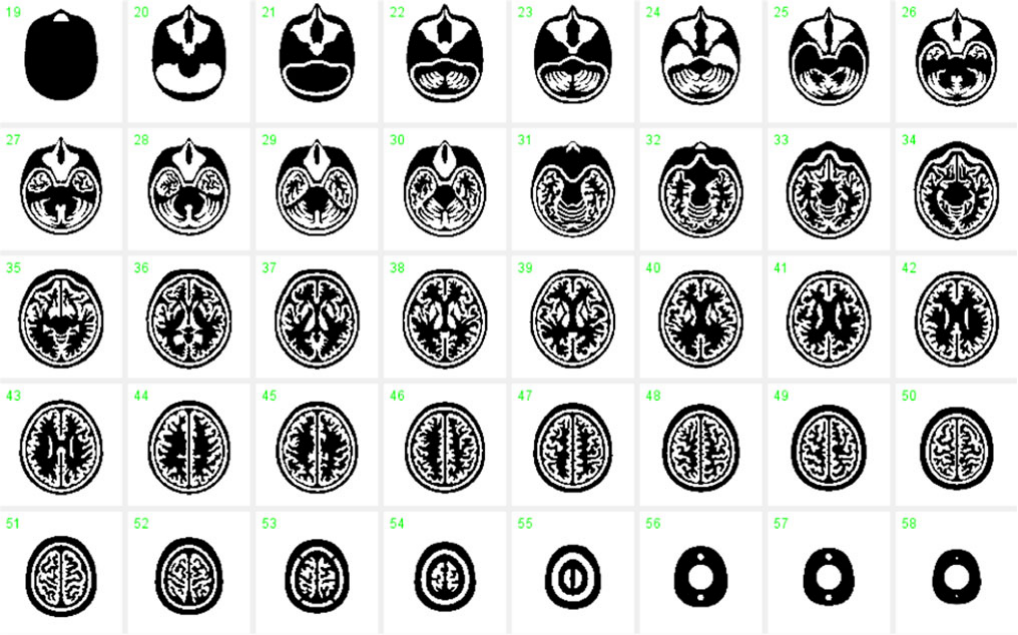
Digital design of the phantom on a 2-dimensional domain. The *areas in white* correspond to the air space, which may be filled with the radioactive solution and K_2_HPO_4_ solution for the grey matter and skull compartments, respectively. The additional air space corresponds to the trachea

The procedures to construct the 3-dimensional phantom are illustrated in Fig. 1. The basic design of the phantom was first generated using a set of T1-weighted MR images of brain obtained from a healthy 26-year-old Japanese male volunteer. The specific sequence included the gradient echo with inversion recovery, which provided gap-less, tomographic images at 1.0-mm intervals. MR images were first segmented using a K-means procedure. Then, the head contour, skull regions, ventricular regions, tracheal air space, and grey and white matter segments were manually illustrated on the computer screen, in a 2-dimensional tomographic domain (Fig. 1a). The grey matter segment included the frontal, parietal, posterior, and temporal cortex areas with the striatum, thalamus, and mid-brain regions. The image size was 440 × 440 pixels (0.5 mm/pixel), and 51 slices were acquired at 3.6-mm axial slice intervals over the whole phantom. The grey matter structure was covered in 34 total slices. During the illustration procedure, a modification was made to remove global distortions in the head and brain structures and to fit a 3-dimensional head model. The tomographic data were then exported to a 3-dimensional computer-aided design (CAD) software (Rapidform2006-sp1, INUS Technology Inc., Seoul, South Korea). The volumetric data were then interpolated in the axial direction (see Fig. 1b). The grey matter and bone compartments were designed so that they could be filled with liquid solutions, and the remaining area, except for the tracheal space, was filled with a polymer resin (see Fig. 2). The grey matter component can be filled with a radioactive solution, and the skull region is typically filled with a bone-equivalent solution of K_2_HPO_4_ as suggested in an earlier study [14]. The white matter region was not to be filled with liquid but was made of a photo-curable polymer, and thus the white matter region became identical to the CSF and scalp. An attention was made to avoid closed areas in the grey matter and bone segments, so that every part of the compartments could be filled with liquid. The radioactive liquid and K_2_HPO_4_ solution can then be diluted into each of the two compartments. In addition, no supporting resin materials were placed both in the grey matter and skull components, although the standard procedure is to place fine pillars to support the fine structure particularly for horizontal wall. The speed of the stereo-lithographic manufacturing process was slowed down, with increased pitch (Fig. 1c). The manufacturing process required whole 3 days to complete. In total, five sets of phantoms were constructed. Of these, two sets were constructed at the same time from the first lot, while the other three were generated from the second lot.

### Evaluation of the phantom

The weight and height of the phantom, as well as volumes of the grey matter and skull compartments were measured for all 5 phantoms. The phantoms were then scanned by X-ray CT using a Symbia T6 CT/SPECT hybrid scanner (Siemens, Chicago, IL, USA) without filling the phantom with liquid. The pixel size of the X-ray CT was 0.5 × 0.5 mm^2^, and the slice pitch was 1.5 mm. The agreement between the X-ray CT and digital design, and the consistency across the five phantoms were evaluated. Additional intention was whether the wall inside the phantom is smoothly constructed or not. Distilled water was mixed with a small amount of detergent and the grey matter compartment of a phantom was filled with it. The skull compartment was filled with the K_2_HPO_4_ solution at the suggested concentration [14]. Both compartments were scanned again using X-ray CT. The X-ray CT images were investigated whether air bubbles remained in the phantom or not.

A transmission scan was carried out on these phantoms using a 3-headed gamma camera (Toshiba GCA-9300, Tokyo, Japan) fitted with 400-mm focal length, low energy, high resolution, symmetric fan beam collimators (N2) from the same vendor. The procedures have been described in our earlier protocol [2, 15, 16] and were shown to provide an accurate attenuation *μ* map of the object [2, 15, 16]. The 132-mm rotation radius used in these studies resulted in a reconstructed field of view (FOV) with a 22-cm diameter [15]. One head was used for the transmission scan, and a 25-cm long ^99m^Tc rod source (74 MBq) was placed at the focal line of its collimator. After a 15-min blank scan, transmission projection data were collected for 15 min. The SPECT cameras were continuously rotated to collect 90 projections over 360° (10 s/projection). The energy window selected was 20 % on 140 keV. After compensating for the radioactive decay of the ^99m^Tc, the inverse of the projection data was multiplied by the blank projection data, to which the filtered-back projection program was applied to reconstruct images of the attenuation coefficients.

### SPECT/PET acquisition

Two sets of SPECT scans were performed on a phantom using a Symbia T6 scanner from Siemens (Chicago, IL, USA) fitted with a low-energy high-resolution (LEHR) collimator set. In the first experiment, the grey matter compartment was filled with a ^99m^Tc solution of approximately 20 MBq, while in the second experiment this compartment was filled with an ^123^I solution of approximately 20 MBq. In both experiments, the skull region was filled with the K_2_HPO_4_ solution at the concentration suggested by de Dreuille et al. [14] (100 g of K_2_HPO_4_ diluted to 67 g of distilled water). The SPECT acquisition followed a standardized protocol for clinical CBF quantitation using ^123^I-iodoamphetamine, as recently described [17]. Seven frames of a dynamic SPECT scan were acquired over a 28-min period at 4 min per frame. The matrix size was 64 × 64, and the number of projection data was 90. Before the reconstruction, all projection data were summed over the whole period, normalized for detector non-uniformity, and calibrated for the centre-of-rotation using the standard vendor software. Then, these data were reconstructed using the QSPECT reconstruction program, including the attenuation correction and scatter correction procedures, as recently described by Iida et al. [17]. A single threshold level (% of the peak of projection data), which was consistent with that in a clinical study, was assigned to define the head contour and generate a uniform attenuation coefficient map. Reconstructed SPECT images were calibrated in Bq/mL, which provides independence from the scanning parameters such as the acquisition time, number of views, matrix size, zoom factor, etc. [17].

Scans were also performed on a phantom using a ECAT ACCEL PET scanner (Siemens-CTI, Knoxville, TN, USA), which provides an intrinsic spatial resolution of 4.5 mm full-width at half-maximum (FWHM) at the centre of the field-of-view (FOV). PET scanning was performed in 2-D mode. Following a 10-min transmission scan using a rotating external ^68^Ge-^68^Ga rod source, 18F-solution of approximately 37 MBq was inserted into the grey matter compartment of the phantom. PET scan was carried out for 10 min. Images were reconstructed using a standard Filtered-Back Projection (FBP) using a Hunning Filter, with standard correction procedures for random, detector normalization, attenuation, and scatter. Eight-millimetre post-filter also applied.

### Data analysis

X-ray CT and SPECT images were registered to the digital design images of the phantom. The agreement with the X-ray CT images was visually analyzed for all five phantoms, particularly the agreement of fine structures of the cortex regions, the presence of residual resin materials in grey matter and bone compartments, and smoothness of the inner phantom surface or local distortion. It was also analyzed whether air bubbles were left in the grey matter or bone compartments on for the experiments filling both compartments with liquid.

Digital design images for the grey matter were extracted and were smoothed using a 3-dimensional Gaussian filter so as to match the spatial resolution of each of SPECT and PET images. The agreement between the SPECT and PET images acquired with the ^99m^Tc and ^123^I, and ^18^F solutions; the digital design images were visually evaluated.

Three ROIs were placed on the attenuation coefficient images obtained from the ^99m^Tc rod source-based transmission scan, namely the brain region, skull region, and whole head contour as carried out in our earlier study on young healthy volunteers [2, 15, 16]. The averaged attenuation coefficient values were obtained for each of these three ROIs and were compared with the values obtained for the young healthy subjects.

All data are presented as the mean ± 1 SD. Student’s *t* test was used to evaluate the difference, and *p* < 0.05 was considered statistically significant.

## Results

The results from the phantom assessment are summarized in Table 1. The volume of the skull component was 306.0 ± 1.9 mL, which was consistent with the expected volume of this component calculated as a summation of the surface area at each slice times the slice interval (305.6 mL). However, the grey matter volume was 562.2 ± 0.9 mL, which is 11.6 mL (2.1 %) greater than the estimated value of 550.6 mL. The phantom height was 212.7 ± 0.1 mm, and the weight of the phantom, excluding the internal liquid, was 1996.8 ± 1.9 g. It is important to note that once the phantom was filled with water, the weight becomes heavier than the original weight. It required more than a day to remove water inside the compartments so that the weight becomes closer to the original value <5 g.

**Table 1:** Physical assessment of five pieces of the 3-dimensional brain phantom. The volume of the bone space essentially agreed with the designed parameter, but the grey matter region had a significantly greater value by 11.6 mL (2.1 %)

	#Phantom	Grey vol (mL)	Bone vol (mL)	Weight (g)	Height (mm)
1	1	564	303	1999	212.80
1	2	562	307	1996	212.80
2	3	563	308	1994	212.62
2	4	562	306	1998	212.69
2	5	562	306	1997	212.67
	Mean ± SD	562.6 ± 0.9	306.0 ± 1.9	1996.8 ± 1.9	212.7 ± 0.1
	Ideal value	550.6 mL	305.6 mL		

**Fig. 3:**
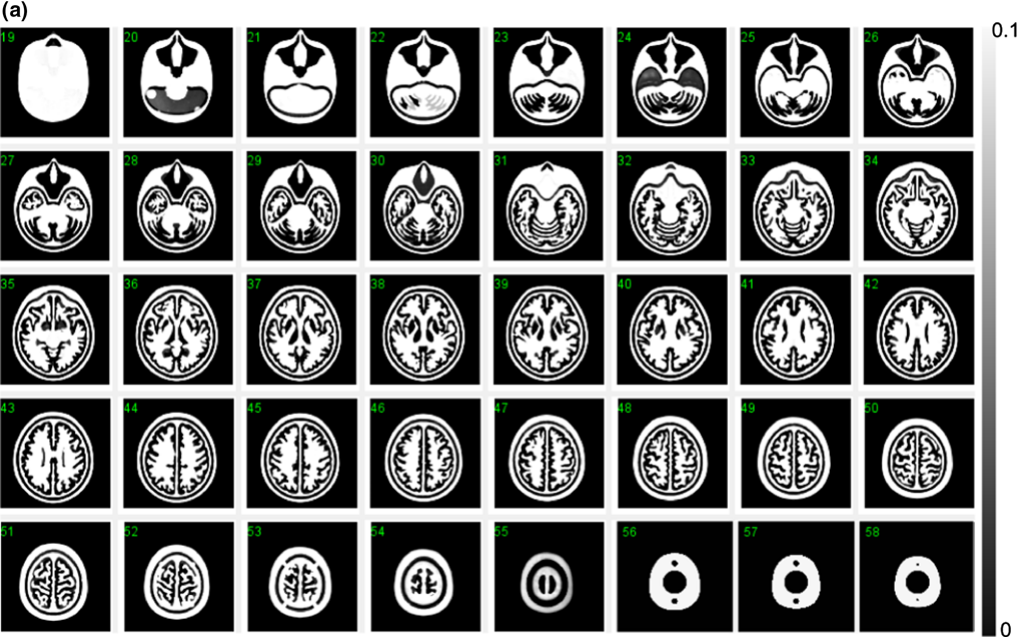

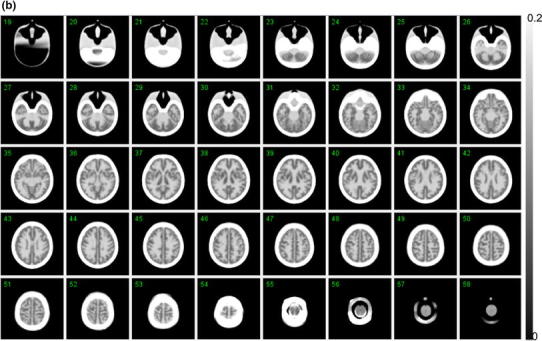
**a** X-ray CT image of the developed phantom, which does not contain water or bone-equivalent liquid inside the phantom. Note that the images are converted to attenuation μ value with a peak value of 0.10 (cm^−1^). The images are aligned to the digital design shown in Fig. 2. No remaining materials or irregular surface are seen inside the grey matter or bone compartments of the phantom. **b** X-ray CT images of a phantom that contains water in the grey matter region, and a K_2_HPO_4_ solution in the bone compartment. Note that the images are converted to attenuation μ value with a peak value of 0.20 (cm^−1^). The images are aligned to the digital design of Fig. 2. There are no air bubbles remaining in the phantom

Figure 3a, b shows example X-ray CT images of a phantom without and with filling liquid into the compartments, respectively. Both images were aligned and re-sliced to match to the digital design images shown in Fig. 2. Note that X-ray CT images demonstrated a high contrast in the bone area due to K_2_HPO_4_ solution, and slightly reduced contrast in the grey matter area compared to the polymer resin areas. The visual analysis showed no particular distortion of the fine structure or resin materials remaining inside the compartments, as were also in other 4 phantoms. It was also confirmed that there were no apparent differences among the five phantoms on the X-ray CT images. No air bubbles were visible in either component.

The results of the attenuation coefficient values measured by the transmission scan using a SPECT camera are summarized in Table 2, along with values observed in healthy young volunteers in a previous study using the same procedures [2]. The attenuation values observed were 0.168 ± 0.006, 0.161 ± 0.006 and 0.206 ± 0.008 cm^−1^, corresponding to the value averaged over the whole head contour at the slice indicating the maximum cross section, the cortical grey matter region and the skull (bone) region, respectively. These values were similar to those from the healthy volunteers determined by the same criteria in our earlier study, which were 0.166 ± 0.01, 0.155 ± 0.007 and 0.197 ± 0010 cm^−1^, respectively.

**Table 2:** Results of attenuation μ values for the averaged brain, cerebral region, and bone region that were obtained with the ^99m^Tc-based transmission assessment

	μ values (cm^−1^)	
	Phantom	Human
Averaged over head	0.168 ± 0.006	0.166 ± 0.010
Cerebral tissue	0.161 ± 0.006	0.155 ± 0.007
Bone	0.206 ± 0.008	0.197 ± 0010

**Fig. 4:**
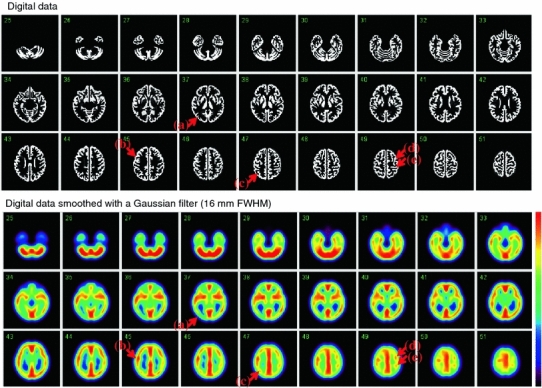
Digital design of the grey matter area of the phantom in the original form (*top*) and after a smoothing operation with a Gaussian Filter of 16-mm full-width at half maximum (*bottom*), which corresponds to the spatial resolution of the SPECT images shown in Fig. 7. Note that the digital design data are noted with unity and null values, corresponding to the grey matter and other regions, respectively

**Fig. 5:**
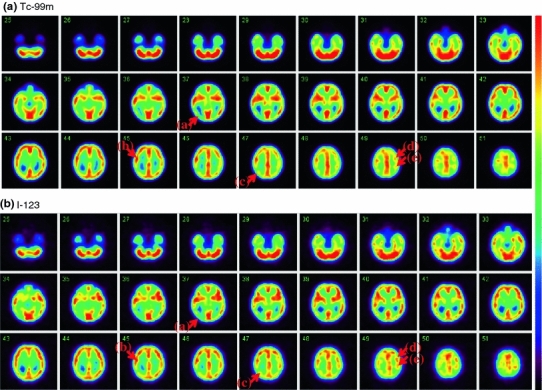
SPECT images of the phantom filled with a ^99m^Tc solution (*top*) and ^123^I solution (*bottom*) in the grey matter compartment and a K_2_HPO_4_ solution in the bone compartment. Both images are aligned to the digital design shown in Fig. 4. SPECT images of the phantom filled with a ^99m^Tc solution (*top*) and ^123^I solution (*bottom*) in the grey matter compartment and a K_2_HPO_4_ solution in the bone compartment. Both images are aligned to the digital design shown in Fig. 4

Figure 4 shows simulated images of the phantom, which represent only the grey matter component, without and with a 3-dimensional Gaussian filter that corresponded to the spatial resolution of the reconstructed SPECT images. Although homogeneous counts are assumed over the entire grey matter regions, the smoothed images (16 mm FWHM) represent inhomogeneous distribution. Figure 5 shows reconstructed SPECT images of the phantom, in which the grey matter component was filled with the ^99m^Tc and ^123^I solution, and the bone compartment was filled with the K_2_HPO_4_ liquid. The images, reconstructed using the QSPECT software including the attenuation, scatter correction using the attenuation coefficient images generated by means of the edge detection on the SPECT image, demonstrated reasonable agreement with the simulated images shown in Fig. 4 for both ^99m^Tc and ^123^I. Detailed structure is identical including the left–right asymmetrical distribution as follows; the counts in the occipital lob region as indicated in (a) are attributed to the thicker structure of the grey matter in this region, and the small spots indicated by (a–d) arrows are attributed to the presence of the cortical fissure. Images were also similar between the ^99m^Tc and ^123^I images.

**Fig. 6:**
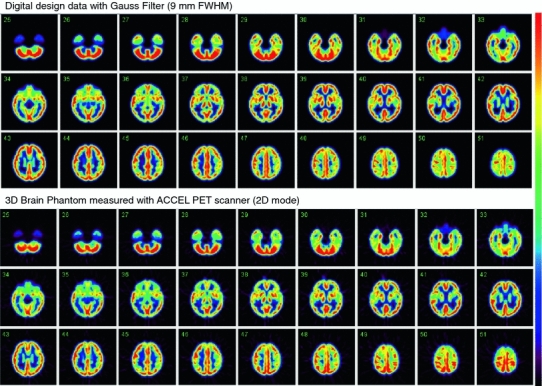
Digital design of the grey matter area of the phantom after a smoothing operation with a Gaussian Filter of 9-mm full-width at half maximum (*top*), and PET images of the phantom filled with a ^18^F solution (*bottom*). PET images are aligned to the digital design shown on *top*

Results from PET experiments are also compared with the simulated digital images in Fig. 6. Images showed good agreement with the digital design after smoothing with a Gaussian filter of 9 mm FWHM.

**Fig. 7:**
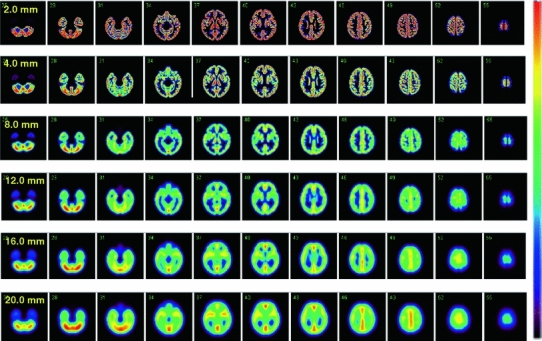
Digital images of the grey matter region of the phantom with various magnitudes of the 3-dimensional Gaussian filter. The magnitude of the filter is given as full-width at half-maximum. Smoothing reduces the contrast, and the magnitude of modification varies at different regions, depending on the structure

Figure 7 demonstrates the effects of applying a different magnitude of 3-dimensional Gaussian filter to the simulated phantom images that contain radioactivity only in the grey matter compartment. A greater amount of smoothing filter resulted in a reduced high-to-low contrast of the phantom images. Note that a particular part of the structure vanishes, and other regions become a cluster, by increasing the FWHM. It can be seen that relative contrasts in the phantom images vary dependent on the magnitude of 3-dimensional smoothing factor, largely attributed to the grey matter structure on the 3-dimensional domain.

## Discussion

This study demonstrated that the photo-curable laser-modelling technique, using the hydrophobic resin, can be used to construct a 3-dimensional brain phantom containing a fine grey matter structure with a detailed head contour and skull-equivalent and trachea components. With careful attention during the manufacturing process, the constructed phantoms appeared to be reasonably reproducible and agreed with the digital design within the accuracy of our image-registration technique. The supporting structures in the inner spaces, which have been the need in typical manufacturing process, could have been well avoided, providing smooth, non-distorted structure inside the grey-matter and skull compartments. The optimized procedures, including the decreased manufacturing speed during the laser-beam construction, pitch selection, and temperature, appeared to be adequate to ensure the solidity and reproducibility of the phantom.

As shown in Table 1, the variation in the volumes was ±0.2 % (or 0.9 mL) and ±0.6 % (or 1.9 mL), corresponding to the grey matter and skull regions, respectively. The total weight and height were also reproducible with variations of ±0.1 % and ±0.05 %, respectively. The attenuation coefficients of the phantom (Table 2), which were obtained using a ^99m^Tc-rod source transmission measurement, were equivalent to those in healthy volunteers, which were assessed using the same experimental procedures in our earlier study for the whole brain, the cerebral tissue area, and the skull area [2]. This is because the photo-curable material used in this study has a similar attenuation coefficient and density (1.07 g/mL) compared to those of the human brain. The X-ray CT images shown in Fig. 3a, b demonstrated a good agreement with the digital design of the phantom shown in Fig. 2. More importantly, there were no residual resin materials on the X-ray CT images, nor any irregular inner wall surface of the phantom in the grey matter or bone compartments. The photo-curable laser-modelling technique usually requires supporting bridges (or pillar structures) when designing structures in the horizontal direction. Our efforts of choosing optimal manufacturing procedures including the reduced speed, temperature and pitch, appeared to be feasible in order to remove such a supporting structure, which is essential in this 3-dimensional brain phantom. It is also important to note that the air bubbles could be well removed relatively easily from both the grey matter and bone compartments due to the connectivity of the liquid space.

In this phantom, we used a bone-equivalent solution of K_2_HPO_4_ as suggested in an earlier study [14]. This solution is known to have similar photon absorption coefficient values with bone or hydroxylapatite for a wide photon energy range, e.g., from 60 to 600 keV. This enables the applicability of this phantom even to the X-ray CT-based attenuation correction assessment. Use of this phantom also for PET acquisition with the energy of 511 keV is also valid. It should be noted that K_2_HPO_4_ is water soluble, and the solution could be concentrated until absorption coefficient values become greater than that of the human bone, while hydroxyapatite is not water-soluble. The water-soluble attenuation material is advantageous.

Of the note is that the SPECT images of the 3-dimensional brain phantom were consistent with the digital design of the phantom for both ^99m^Tc and ^123^I, after applying a Gaussian filter that corresponded to the spatial resolution of the reconstructed images. This is attributed to the accurate reconstruction software (QSPECT) [2, 17], including adequate attenuation- and scatter-correction procedures, employed in this study [2, 17]. The identical images between the ^99m^Tc and ^123^I isotopes further suggest the adequacy of the compensation procedures for penetration from high-energy photons emitted from the ^123^I isotope itself [2, 17]. PET images reconstructed with the standard FBP procedures including the detector normalization, random, attenuation, and scatter correction processes were in a good agreement with the smoothed digital design images.

This phantom may help evaluating the quality of clinical PET and SPECT brain images assessed with a variety of equipment installed at different institutions. There are several error factors that may cause distortion or irregular radioactivity distribution in the reconstructed images, attributed to imperfect attenuation- or scatter-correction procedures and/or other physical error sources. Quantitatively assessing these factors are often important in multicenter-clinical trials using existing SPECT and PET devices. Recently, Joshi et al. [12] proposed to assess the cross-consistency of PET images from different institutions using the Hoffman 3-dimensional brain phantom. The 3-dimensional brain phantom presented in this article may be better suited for such purpose. Joshi et al. [12] demonstrated that errors introduced by the attenuation and scatter correction procedures are significant and vary depending on the PET device. They also demonstrated that these errors are very different for human brain as compared with those for the Hoffman 3D brain phantom, because of the cylindrical shape of the Hoffman brain phantom with no skull or neck in the Hoffman 3D brain phantom. The phantom developed in this study might be better suited for determination of the attenuation and scatter correction errors for PET in multicentre studies. Further, advantage of the present phantom could be in the usage of evaluating brain SPECT images. Brain SPECT reconstruction often employs a software-based head contour detection for generating an attenuation coefficient distribution, as also done in QSPECT software. The present phantom is suitable in order to evaluate adequacy of the head-contour detection algorithm. Additional error factor could be due to the contribution of radioactivity outside of FOV. This might be evaluated by placing additional radioactivity source that simulates the clinical distribution for each radiotracer. Further systematic studies would be needed in order to confirm the real contribution in multicenter studies using SPECT.

The photo-curable polymer has been known to absorb water into the polymer materials and thus could alter the volumes. However, a recently developed water-repellent epoxy-resin (TSR-829) [13] seemed to be an appropriate material for the phantom, as there was minimal dilution of the water-liquid into the resin. The persistence of water in the grey matter and bone compartments after removing the water-liquid is attributed to the surface tension of water. The total weight of the phantom could become the original value when removing the water from the phantom, after 1 day of dry. Although further careful investigations are needed, the results in the present study suggest that this phantom is an adequate tool to evaluate PET/SPECT image quality.

A systematic discrepancy of 11.6 mL (approximately 2 %) was observed in the grey matter volume compared to the digital design. This could have occurred during the axial interpolation in the CAD procedures. Note that the digital data are given as tomographic data at 3.6-mm intervals, and a 3-dimensional interpolation was done to establish the slice-to-slice connectivity. Therefore, the digital data are not necessarily identical to the actual structure of the constructed phantom. However, the difference was not clearly visible in the X-ray CT images compared with the digital design. Intrinsic differences should be present in the precise structure and volume of the constructed phantom, but the reproducibility of the phantom is probably a more important issue. Further investigation is needed.

One drawback of the present 3-dimensional brain phantom compared to the Hoffman 3-dimensional phantom is that there is no structure corresponding to the white matter region. It is theoretically possible to insert small structures in the white matter regions, but additional work will be required to establish a liquid flow in these regions. In addition, air bubbles may be difficult to be removed, requiring further technical challenges.

The reconstruction program used in this study was a QSPECT program and was recently evaluated in a multicenter study [17]. The study demonstrated the adequacy of quantitating cerebral blood flow images both at rest and after acetazolamide treatment in clinical institutions, but further evaluations of the physical accuracy and cross-institutional consistency of reconstructed images are required among the participating institutions. The present 3-dimensional brain phantom might be valuable for this purpose, particularly when data are obtained with a variety of clinical settings and equipment. These studies are highly desired in the future.

A recent report also suggested the impact of utilizing the SPECT device in multicenter clinical trials. This is based on the physical features of the SPECT equipment, namely that the magnitude of the two major sources of attenuation and scatter errors is independent of the scanner in SPECT, unlike in PET. Scatter and attenuation occur in the object and are thus object dependent, but are not dependent on the geometry of the imaging equipment [18]. Therefore, once a software program is developed to provide accurate image reconstruction with compensation for both attenuation and scatter, the program should be able to provide quantitative images that are intrinsically independent of the geometric design of the SPECT cameras. This is an attractive feature of SPECT for multicenter clinical studies. The present 3-dimensional brain phantom would be valuable in order to verify this concept.

## Conclusion

A 3-dimensional brain phantom containing the grey matter, skull, and tracheal structures with a realistic head contour has been developed using a laser-modelling technique with a photo-curable polymer (transparent TSR0829; 1.07 g/mL). The high consistency with digital data and reproducibility across constructions suggest that this phantom can be used for multicenter evaluations of PET and SPECT images.
